# Dr. Coe and the Octopus Management

**Published:** 1908-02

**Authors:** 


DR. COE AND THE OCTOPUS MANAGEMENT.—I never make a statement that I can not verify. In a recent editorial, I said of Dr. Henry Waldo Coe, of Portland, Oregon, editor *Medical Sentinel* (in speaking of the outrage of the Octopus on the medical press, excluding all but Octopus journals from the exhibit hall) * * *

"And had not the recalcitrant, but now recidivous Coe, who was looked to to second the "Red Back's" protest, and lead a revolt of the medical editors, joined in the unanimous approval of the Octopus' management, I would be tempted almost to hope and say that the medical profession wouldn't stand for such treatment (as was accorded Dr. Abbott by the Octopus), but would demand a square deal. They will stand for *anything*, at the hands of the Monster Octopus."

In the December issue of the *Medical Sentinel*, Dr. Coe challenges me to point out "when and where" he "joined in the unanimous approval of the Octopus Management."

Here it is: Pages 2057, 2058, 2059 of the *Journal American*

*Medical Association,* June 15, 1907 (official stenographic report of the discussion that followed the reading of the financial report of the trustees, and the Secretary-Editor). It is worse than I thought it was; he seconded the motion. Dr. Coe had been severely criticising the several items in the report, and said:

"Under Exhibit E, they have an inventory of paper, stock, metal, type, buttons, ink and coal, amounting to $14,336.59, which, of course, are assets, according to the Board of Trustees. I contend that these are not assets, and that this exhibit is not made to show the real condition of affairs, but is intentionally misleading, and—

"The President: Men's names and men's motives are not debatable; but the acts of men and the consequences of their acts are debatable."

At the close of the discussion (stenographer's report—continued):

"Dr. L. S. McMurtry, Kentucky: I move that the House of Delegates tender a vote of confidence and thanks to the Secretary-Editor and Manager for his faithful, honest and efficient services.

"This motion was seconded by several, but Dr. Coe said that, inasmuch as he had started this discussion, but had said nothing against the Editor and was very careful not to do so, he took great pleasure in seconding the motion.

"Dr. Hubert Work, Colorado, moved to amend that the Board of Trustees be included in the vote.

"The amendment was seconded, accepted and the original motion as amended was unanimously carried."

